# Lassa virus live tracking and lineage assignment: how nextstrain can enhance surveillance and public health in Africa and beyond

**DOI:** 10.1080/22221751.2026.2640699

**Published:** 2026-03-04

**Authors:** Richard Olumide Daodu, Jennifer Chang, Joseph B. Prescott, Knut Reinert, Denise Kühnert

**Affiliations:** a Center for Artificial Intelligence in Public Health Research, Robert Koch Institute, Berlin, Germany; b Department of Mathematics and Computer Science, Freie Universität Berlin, Berlin, Germany; c Vaccine and Infectious Disease Division, Fred Hutchinson Cancer Center, Seattle, WA, USA; d Center for Biological Threats and Special Pathogens, Robert Koch Institute, Berlin, Germany; e Max-Planck-Institute for Molecular Genetics, Berlin, Germany

**Keywords:** Lassa virus, Nextclade, Nextstrain, CLASV, genomic surveillance, epidemiology, phylogenetics

## Abstract

Lassa virus (LASV), a zoonotic, bi-segmented arenavirus endemic to West Africa, causes seasonal epidemics with substantial morbidity and mortality. Increasing frequency of exported cases emphasizes the need for near real-time genomic surveillance to support outbreak response and clinical decision-making. We developed a suite of open-access resources on the Nextstrain and Nextclade platforms tailored to LASV. These include live phylogenetic and phylogeographic visualization, as well as Nextclade builds for rapid mutation detection and lineage assignment based on the L and S segments and the glycoprotein complex (GPC). A dedicated GPC phylogeny enables tracking of clinically relevant mutations, including immunologically relevant variants such as alanine at position 76 (A76), which is prevalent in lineage II and is implicated in reduced binding of monoclonal antibody 25.10C. The Nextclade tools distinguish LASV from other mammarenaviruses and assign lineages with Matthews correlation coefficients exceeding 85%. These tools are available at https://nextstrain.org/lassa for immediate use in surveillance, data annotation, outbreak response, and potentially support clinical decision-making in both endemic regions and exported-case scenarios. A key limitation is dependence on genomic data quality and recency. Although sampling-to-submission delays have decreased over the past 40 years, the average delay in the past five years remains ∼2 years. In many endemic LASV regions, challenges, such as limited resources, infrastructure, and previous experiences of stigmatization and political repercussions linked to outbreak reporting, restrict data sharing. The resulting disparities and delays may hinder comprehensive surveillance and timely response, with implications for global public health.

## Introduction

The genome of an organism is its molecular fingerprint. When sequenced, this genetic information can reveal several phenotypic characteristics. In pathogens, specific traits such as virulence, immune evasion, and drug resistance are often associated with defined genomic variants [1–3]. Once experimentally validated, mutations linked to these traits can reliably predict pathogen characteristics and clinical outcomes [3]. Furthermore, comparative genomic analysis using well-curated databases enables the reconstruction of evolutionary history, identification of likely origins of infection, and tracking of outbreak dynamics and origins. These insights collectively support improved outbreak containment, patient management, therapeutic development, and the formulation of evidence-based public health strategies.

Recent advances in pathogen genomics and near real-time bioinformatics have transformed outbreak surveillance and response. The Nextstrain platform offers live phylogenetic visualization through an interactive web interface [4]. It integrates continuously updated public genome data into live phylogenetic trees, enabling users to observe lineage dynamics and mutation patterns across time and geography. During the SARS-CoV-2 pandemic, Nextstrain proved to be a vital tool for public health authorities, facilitating early detection of divergent strains and localized outbreak monitoring [5–8].

**Figure 1.**
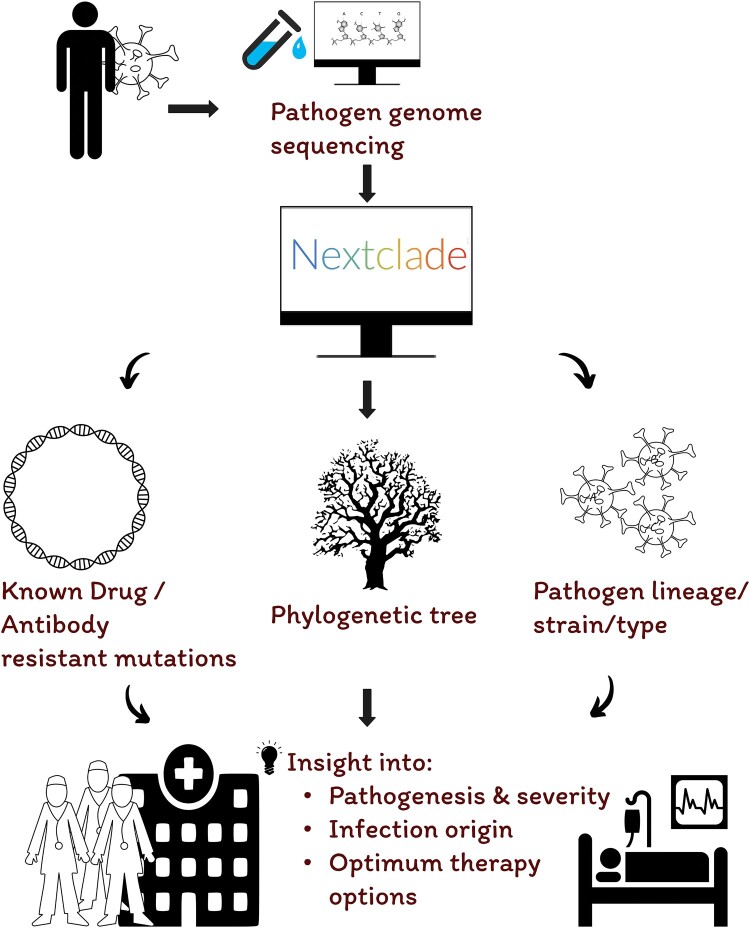
Potential clinical application of Nextclade for pathogen genomic analysis. Note: A patient presenting with a suspected infection undergoes diagnostic testing, and a clinical sample is collected for pathogen genome sequencing. The resulting sequence is analyzed using the Nextclade web platform, which rapidly performs mutation calling, lineage assignment, and phylogenetic placement. This streamlined process provides actionable genomic insights such as the presence of resistance-associated mutations or lineage-specific traits that can support clinicians in selecting the most appropriate therapeutic strategies and contribute to informed public health responses.

Complementing Nextstrain’s live trees, the Nextclade platform provides per-sample genomic analysis [9]. Accessible via clades.nextstrain.org, it allows users to upload one or more viral genomes for mutation calling, quality control, lineage assignment, and placement on a standardized reference tree. This rapid and reproducible analysis framework makes Nextclade a practical tool for genomic surveillance and clinical diagnostics. Although widely adopted during the SARS-CoV-2 pandemic [10,11], its broader application to other high-risk pathogens – especially those causing recurrent seasonal epidemics like Lassa virus – remains underutilized. Its potential use in clinical decision-making is illustrated in Figure 1.

Lassa fever (LF) is a severe viral hemorrhagic illness endemic to West Africa, with an estimated 300,000 infections and up to 5,000 deaths annually [12]. The overall fatality rate is relatively low (∼1%) [12], but is much higher among pregnant women and fetuses [13–16]. The disease is caused by Lassa virus (LASV), a member of the family *Arenaviridae*, and its primary reservoir is *Mastomys natalensis*, with infections increasingly reported in additional host species [17,18]. The clinical features are comparable to those observed in infections caused by other arenaviruses, such as Junin virus, the causative agent of Argentine hemorrhagic fever [19]. They are grouped as Risk Group 4 pathogens – with experimentation with viable viruses limited to maximum Biosafety facilities. To date, no licensed vaccine exists for LF. While LASV continues to cause yearly outbreaks in West Africa [12,20], exported cases have been reported on multiple continents [21], including Europe and, most recently, China [22]. These occurrences place emphasis on the urgent need for rapid diagnostics, effective therapeutics, and globally integrated biosurveillance systems. Recognizing the urgent need for countermeasures, the World Health Organization (WHO) has designated LASV a priority pathogen for accelerated research and development [23].

The LASV genome is bi-segmented RNA, comprised of a large (L) and a small (S) segment, and encodes four essential proteins: the glycoprotein complex (GPC) precursor, nucleoprotein (N), matrix protein (Z), and RNA-dependent RNA polymerase (L). The GPC undergoes post-translational cleavage into three subunits: the stable signal peptide (SSP), glycoprotein subunit 1 (GP1), and glycoprotein subunit 2 (GP2). As the sole surface-expressed protein, the GPC is responsible for mediating host cell entry, making it a prime target for vaccines and therapeutics [20,24]. This also makes tracking its evolution and mutations specifically important.

Genomic analyses have delineated seven distinct LASV lineages with largely non-overlapping geographic distributions: lineages I–III in Nigeria [25]; IV in Sierra Leone, Guinea, and Liberia [26]; V in Mali and Côte d’Ivoire [27]; and VII in Togo and Benin [28,29]. The strain detected in *Hylomyscus pamfi* in Nigeria was assigned to lineage VI [17]. These lineages differ not only genetically but also in clinical severity and immunogenicity [26,30–33]. For example, lineage VII has been shown to be more pathogenic than lineage II in animal models [33], and neutralizing antibodies are generally more effective against the lineage from which they were derived [31,32]. Together, these observations clearly suggest that identifying the LASV lineage causing disease – whether in an individual patient, an epidemic, or an imported case – can immediately inform likely geographic origin, anticipated severity, and the potential utility of lineage-specific antibody therapeutics. These factors make rapid and accurate lineage assignment increasingly important for guiding clinical management and for informing therapeutic strategies and public health response.

Despite the recurring nature of LF outbreaks and the expanding availability of next-generation sequencing (NGS) technologies across Africa [34], genomic investigations often rely on manually curated phylogenetic pipelines. During LF outbreaks, lineage assignment is typically performed using traditional phylogenetic approaches [22,26,35], which require substantial LASV-specific and phylogenetic expertise and are not easily scalable during public-health emergencies. We have bridged this gap with CLASV, a machine-learning tool based on the LASV glycoprotein gene (GPC) [36] but complementary, online, easy-to-use tools are still needed to support near real-time genomic surveillance. As of this writing, most LASV sequences submitted to public databases (e.g. NCBI Virus) remain unannotated for lineage, delaying interpretation of reconstructed LASV phylogenies and hampering rapid public-health responses, which we address in this study. Meanwhile, the Africa Centres for Disease Control and Prevention (Africa CDC) has identified data barriers to pathogen genomics [37]. These barriers include governance, infrastructure, workforce development, bioinformatics solutions, internet bandwidth, data quality, and data standardization. During the COVID-19 pandemic, social and political stigma and concerns about travel restrictions were also linked to delays in reporting variants of concern [38,39]. Because our surveillance tools depend on timely data sharing and adequate sequencing, we examined how these limitations may have shaped the current LASV dataset in GenBank.

The current therapeutic landscape for LF remains limited. Ribavirin continues to be the most commonly used antiviral, despite longstanding concerns over its toxicity and inconsistent clinical effectiveness [40,41]. Passive immunotherapy, including the use of convalescent plasma or immune serum, has demonstrated variable success since its early applications [42,43]. Notably, antibodies or plasma derived from individuals infected with closely related LASV lineages tend to show greater neutralizing activity [31,32], supporting the importance of lineage-specific immune targeting. More recently, a monoclonal three-antibody cocktail targeting the LASV GPC provided protective efficacy in non-human primates challenged with lineages I–IV [44]. However, its efficacy was significantly lower against lineage VII [45], likely due to escape mutations in the glycoprotein that impair antibody binding. These findings emphasize the urgent need for platforms that can identify and track immune escape mutations and inform lineage-specific therapeutic strategies.

In this article, we present two complementary tools to support interactive and rapid genomic surveillance of LASV. First, we developed an open-source, continuously updated Nextstrain live build for trees (L, S, and GPC phylogenies) that enables interactive visualization of LASV genomic epidemiology, built on curated, lineage-annotated sequence data. Second, we created customized Nextclade builds (L, S, and GPC) optimized for rapid LASV lineage assignment and mutation detection. We extensively validated the Nextclade tools for their ability to distinguish LASV sequences from other mammarenaviruses and to assign known LASV lineages. Together, these tools allow researchers, clinicians, and public health officials to track viral diversity, detect emerging variants, and place new cases within a broader phylogenetic and geographic context. By providing both interactive and rapid analytics and structured genomic reference data, these resources enhance outbreak preparedness, accelerate response efforts, and support clinical and epidemiological decision making.

## Method

### Genomic surveillance patterns of LASV

To visualize the status quo of the data which our tools would be based on, we downloaded all LASV sequence metadata from NCBI Virus (available at https://www.ncbi.nlm.nih.gov/labs/virus/vssi/#/) on 30 September 2025, excluding laboratory and environmental strains (n = 2,761). We retained records with a non-missing sampling date (“Collection_Date”; n = 2,556) and to minimize noise, we removed sequences shorter than 500 nt (less than half the length of commonly sequenced LASV genes GP, NP, and L), yielding 2,333 sequences for analysis. Partial dates were harmonized to concrete timestamps as follows: For collection dates, year only entries were imputed to the last day, 31st December of that year, while year–month were set to the last day of that month. For release dates, year-only entries were imputed to 1st January for that year, while year–month entries were set to the first day of that specific month. In all scenarios, full dates were parsed as provided.

For each record, we computed time-to-release as (Release_Date − Collection_Date) in days and expressed it in years (days/365.25). We visualized delay versus sampling date with a scatter plot and an ordinary-least-squares (OLS) trend line, and summarized delays within fixed sampling windows (2020–2025, 2015–2025, 2005–2025, and all time) by reporting the number of sequences, mean delay, and median delay. Using NumPy [46], we fit an OLS model of delay (years) versus sampling date (encoded as Unix seconds) and reported the slope (years of delay per calendar year) and the coefficient of determination (R-squared). R-squared (R²) was obtained by comparing how much variation in observed delays remains after the model’s predictions (the “unexplained” variation) to the total variation around the average delay; it equals one minus that ratio, reflecting the share of variability captured by the linear trend with an intercept.

From the 2,333 entries, we summarized host species and sampling locations across the full time span. Missing values for host or country were retained and labelled “Unknown” after basic normalization (trim blanks/whitespace). Visualizations were done using Plotly (available at https://plotly.com/python/) and data handled using pandas (available at https://pandas.pydata.org/).

### The Nextstrain LASV realtime workflow

LASV, like other arenaviruses, possesses a bi-segmented RNA genome composed of the L and S segments [20]. Due to the potential for reassortment and recombination between segments, which may bias evolutionary analyses, it is standard practice to analyze each segment independently [25,26]. Furthermore, because the glycoprotein complex (GPC) is the only surface-exposed protein and a principal target of the host immune system and immunotherapies [24], it warrants focused phylogenetic analysis.

We adapted the standard Nextstrain workflow [4] to LASV. The aim is to maintain three LASV live phylogenetic and phylogeographic trees for rapid surveillance. Sequence data and metadata were obtained from GenBank/NCBI and cover the L- and S-segments and the GPC, from the virus’s discovery in 1969 to the present. We parsed and cleaned basic fields (collection date, country, host species, segment), and kept records with missing values to preserve coverage. We standardized title casing and author abbreviations, applied geolocation rules, and added curated annotations, producing harmonized metadata and per-segment FASTAs. The curated, segment-separated inputs (L, S, GPC) are stored as compressed artifacts in cloud storage and are automatically retrieved by the phylogenetic workflow for reproducible builds. The pipeline is orchestrated with GitHub Actions and Snakemake, which run on a regular schedule (currently every 24 h), refreshing inputs from GenBank/NCBI and regenerating the Nextstrain builds.

Sequences were filtered with Augur [47] to exclude laboratory hosts, apply inclusion/exclusion lists (problematic accessions and sentinel inclusions), and enforce minimum lengths of 5,000 nt (L), 2,000 nt (S), and 800 nt (GPC). Alignments were generated with Augur against bundled LASV references (L: NC_004297; S: NC_004296). For GPC, we aimed to ensure codon alignment – as LASV is usually divergent and often requires manual curation [18] – by aligning new sequences onto an existing curated GPC alignment seeded by a prebuilt GPC coding sequence alignment (reference NC_004296.1:c3347–1872).

Using Augur via its application programming interface (API) [47], which wraps multiple tools, we inferred phylogenies in a reproducible Snakemake pipeline [48] integrated with Nextstrain [4]. Maximum-likelihood trees were built with IQ-TREE [49] using the general time-reversible (GTR) nucleotide substitution model. Time-scaled trees were produced with TreeTime [50] via the Augur API, using midpoint rooting and a fixed clock rate of 0.00078 substitutions/site/year [25], with date_inference = marginal, coalescent = opt, and date-confidence estimation enabled. Downstream analyses used Augur [47] to annotate mutations, reconstruct ancestral states, and export outputs to Auspice [4] for interactive visualization. We reconstructed the phylogenetic trees and annotated the clades as described previously [36] and following the appropriate literature [17,25–29]. All seven LASV lineages (I–VII) were represented, with IV and V grouped per Whitmer et al. [28], which noted that their genetic distance is comparable to that observed among sublineages rather than fully separate lineages. As part of the LASV Nextstrain pipeline, new sequences from GenBank are automatically assigned to lineages using the Nextclade dataset (see Methods and Results) before display in the live trees. To highlight potential reassortment, we provide a public Nextstrain tanglegram linking matched isolates between the L- and S-segment trees. All figures were refined using Figma (https://www.figma.com/).

### LASV Nextclade lineage assignment and build design

To enable rapid LASV lineage assignment and mutation calling, we developed three Nextclade [9] builds – in addition to the Nextstrain builds – covering the GPC gene and the S and L segments. The general approach follows past work [51].

To ensure the Nextclade v3 builds classify only LASV, we optimized the seed-cover gate applied before alignment. Nextclade indexes fixed-length k-mers (“seeds”) from each segment’s reference sequence and, for any query, computes the seed cover (fraction of required seeds present, 0–1). The “ – min-seed-cover” threshold is a monotonic gate: a sequence passes if its seed cover is at least the threshold and fails otherwise. Increasing the threshold raises stringency and preferentially filters out off-target or highly divergent sequences.

**Figure 2.**
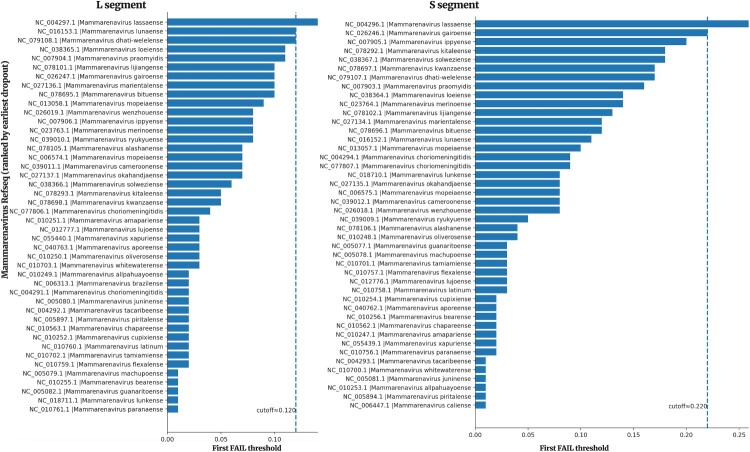
Output from the sweep across mammarenavirus segments to identify the optimal minimum seed-cover cutoff. Note: The chosen cutoffs are visible in the plot (0.120 for L, 0.220 for S). Notably, Luna virus was the last non-LASV to be excluded for the L segment, and Gairo virus was the last to drop for the S segment.

For each LASV segment (L and S), we assembled a background of all mammarenavirus RefSeq sequences for that segment (NCBI taxid 1653394) because other mammarenaviruses are LASV’s closest genetic relatives. We also included the LASV RefSeq used by the Nextclade datasets (L: NC_004297.1; S: NC_004296.1). We then performed a parameter sweep of “ – min-seed-cover” from 0.01 to 0.30 (step 0.01), ran Nextclade against the corresponding LASV dataset, and recorded per-sequence status. Our selection rule was stringent: select the lowest threshold at which the segment’s LASV reference still passes while every non-LASV mammarenavirus fails (Figure 2). This yielded a data-driven operating point that enforces LASV-only acceptance for subsequent processing.

The optimal thresholds were 0.120 for the L segment (NC_004297.1 retained; all non-LASV excluded) and 0.220 for the S segment (NC_004296.1 retained; all non-LASV excluded). The S-segment cutoff was also used for the GPC build. These thresholds were adopted in the deployed online builds. We curated the L and S Nextclade builds (data composition) to mirror their corresponding Nextstrain phylogenies as of 10 October 2025 (see Results). Because the Nextstrain GPC tree is substantially larger, the GPC Nextclade dataset was a curated subset (extending the panel used in Daodu et al. [36]) with representatives for lineages I and VI added (KM822128 and KT992425, respectively). The builds are designed to assign lineages to I, II, III, IV/V (per Whitmer et al. [28]), VI, and VII.

### Reference alignment, scaffold tree, and LASV Nextclade dataset packaging

From these representatives, we generated a reference multiple alignment and a scaffold (reference) phylogeny for each segment (and the GPC gene), then packaged them in the standard Nextclade dataset format and released them via GitHub (see Data Availability). When a query sequence is submitted, Nextclade aligns it to the segment reference, calls nucleotide and amino acid mutations, and assigns a lineage based on mutation profiles and phylogenetic proximity to defined clades on the scaffold tree [9].

### Outputs and usage

Nextclade reports per-sequence lineage assignment, amino-acid substitutions, and the placement of each query on the reference tree. Trees can be interactively coloured by lineage, country, or clinically relevant mutations.

### LASV Nextclade validation dataset and specificity testing design

**Figure 3.**
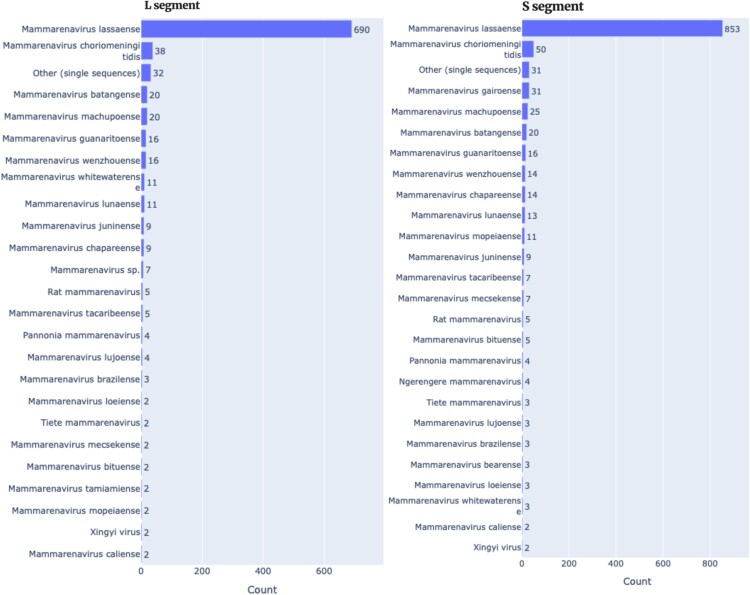
Distribution of the comprehensive test data. Note: This figure summarizes the benchmark corpus used to evaluate species discrimination and lineage assignment. The set is dominated by LASV sequences and includes a broad panel of other mammarenaviruses (NCBI TaxID 1653394), notably other Risk Group 4 arenaviruses such as Guanarito, Junín, and Machupo, among others. Sequences were sourced from NCBI Virus with the filters (≥600 nt, 25 September 1900-27 September 2025, and recent NCBI Virus segment-specific criteria for L and S). Performance for LASV discrimination and initial lineage assignment was evaluated directly on this dataset.

LASV false positives could lead to unnecessary patient isolation, inappropriate treatment, and unwarranted public health alarm, while false negatives could result in missed diagnosis with subsequent transmission and potential death. Hence, we tested our Nextclade builds exhaustively in two scenarios: (i) the ability to distinguish LASV sequences from other mammarenaviruses (and, by extension, other viruses), and (ii) the ability to assign lineages accurately. We downloaded all mammarenaviruses (NCBI TaxID 1653394) from NCBI Virus using these rules: length ≥600 nt; collection dates from 25 Sep 1900 to 27 Sep 2025 (which ensures sampling date); application of NCBI Virus segment-specific criteria for L and S. This yielded a broad mammarenavirus panel for specificity testing, including Risk Group 4 arenaviruses (e.g. Guanarito, Junín, Machupo) alongside LASV. Figure 3 shows the distribution of the comprehensive test data by segment. For the S segment, the dataset contained 853 LASV sequences plus representatives of 55 other mammarenaviruses (total = 1,096; Figure 3; accompanying GitHub data). For the L segment, it contained 690 LASV sequences plus representatives of 55 other mammarenaviruses (total = 916; Figure 3; accompanying GitHub data). Each segment-specific set is LASV-dominant yet includes a diverse panel of non-LASV mammarenaviruses for stringent specificity testing.

We evaluated the three LASV Nextclade builds (L, S, and GPC) alongside our previously published LASV GPC amino-acid classifier, CLASV [36], in both its general and out-of-distribution (OOD) modes. CLASV is a random forest model that classifies sequences into lineages II, III, IV_V, and VII. It uses the Nextclade command-line interface (minimum seed cover 0.01) for rough filtering, GPC extraction, and alignment before sequence encoding for model input. Thus, general CLASV performance at distinguishing LASV from non-LASV is measured as the fraction of sequences labelled “inconclusive” plus those filtered out by Nextclade. The CLASV OOD evaluation measures the model’s ability to label non-LASV sequences as inconclusive rather than incorrectly assigning them to LASV lineages.

### LASV Nextclade validation

We evaluated the live Nextclade builds using curated mammarenavirus datasets. The L-segment set (916 sequences; Figure 3) tested the L build, and the S-segment set (1,096 sequences; Figure 3) tested both the S build and the GPC build. For comparison, we also evaluated CLASV (Daodu et al. [36]; version 1.0.6). Species labels were taken directly from GenBank names/metadata and treated as ground truth.

For each tool, a sequence labelled LASV that received any LASV lineage assignment was counted as a true positive (TP); an LASV-labelled sequence without a lineage assignment was a false negative (FN). A sequence labelled as a non-LASV mammarenavirus that received no LASV lineage was a true negative (TN); if such a sequence was assigned a LASV lineage, it was a false positive (FP). From these outcomes we computed accuracy, precision, recall, F1, and Matthews correlation coefficient (MCC), prioritizing MCC because it is robust to class imbalance.

The metrics are defined as follows:

- Accuracy = (TP + TN)/(TP + FP + TN + FN)
- Precision (“among sequences the tool called LASV, how many truly were LASV”) = TP/(TP + FP)
- Recall (sensitivity; “among true LASV sequences, how many the tool found”) = TP/(TP + FN)
- F1 = 2·(precision·recall)/(precision + recall)
- MCC = (TP·TN − FP·FN) / √[(TP + FP)(TP + FN)(TN + FP)(TN + FN)], a balanced measure that remains informative under the LASV-heavy class distribution.

For an initial assessment of lineage-assignment accuracy using the mammarenaviruses dataset, we grouped the outputs from the S-based tools (S and GPC Nextclade builds) and CLASV into a single dataframe in which each row is a test sequence and each column is a tool’s lineage call. If a sequence was not classified into any lineage (e.g. non-LASV), the cell was set to “unknown/none.” Ground-truth lineages for GPC were derived from the live Nextstrain tree, whose lineage branches were annotated from the literature; in practice, nodes falling within a given lineage clade were labelled with that lineage. To ensure a fair comparison among GPC Nextclade, S Nextclade, and CLASV, we excluded sequences for which only the S-segment nucleoprotein (NP) was available (i.e. S had a lineage call but GPC and CLASV, which require GPC, did not). We then filtered to entries with ground-truth labels and calculated percent correctness for each tool and lineage (see Results). We applied a similar procedure for the L-segment: ground-truth labels were taken from the L Nextstrain live build, and performance was compared to lineage assignments from the L Nextclade tool.

We further performed more comprehensive validation of the S- and GPC-based datasets because the S segment is immunologically and clinically important [20,24] and has more available sequence data than the L segment. We extracted additional sequence IDs from the GPC live Nextstrain tree that were not present in either the S-segment or GPC Nextclade datasets, yielding 342 sequences (Figures 8E and F), which we downloaded from GenBank and used to further validate lineage assignment by the GPC and S Nextclade tools. The annotated GPC Nextstrain build described above, with lineages curated from the literature, was used as the ground truth to evaluate assignment accuracy for both tools.

## Results

### A view of the current LASV genomic surveillance patterns

**Figure 4.**
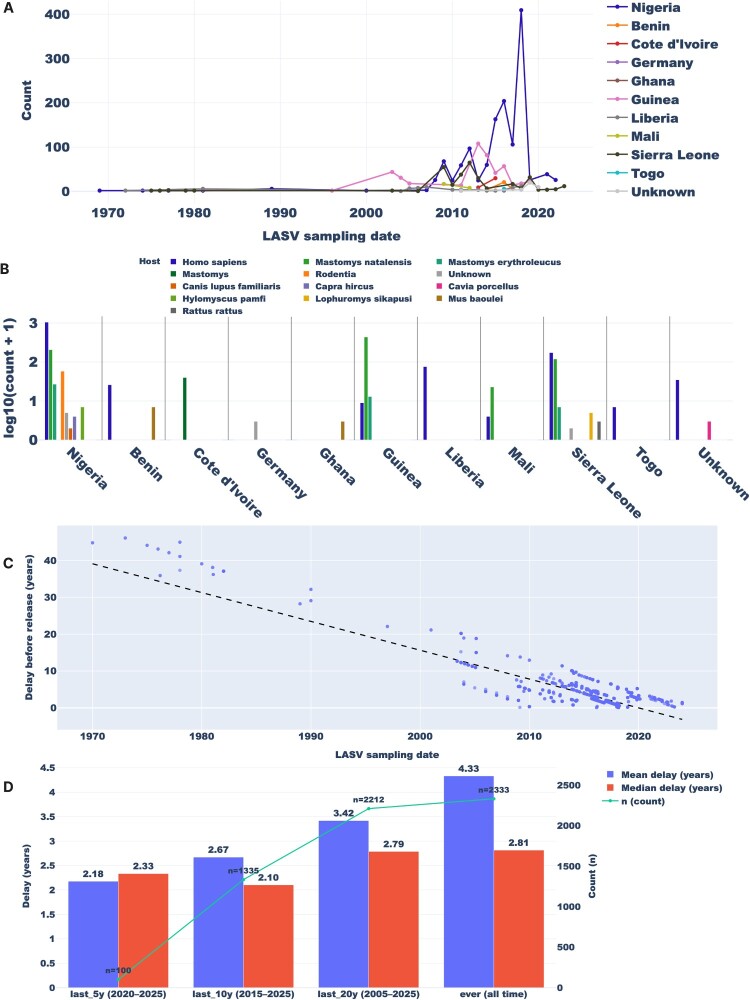
Sampling pattern of LASV (as of 30 September 2025). Note: (A) Temporal distribution. Nigeria shows the longest sampling range, beginning in 1969, the year of the first confirmed LF case. Across countries, submissions drop sharply after 2020, likely reflecting diversion of surveillance resources during the COVID-19 pandemic. Records missing the sampling country are retained and labelled as “Unknown.” (B) Host distribution. Counts are shown on a logarithmic scale. Most sequences derive from humans and *Mastomys natalensis*, the primary rodent reservoir of LASV; other hosts are represented at much lower frequencies. Records with missing host or country are retained and labelled “Unknown” (rendered in grey in the plots). (C) Sampling-to-release delay over time. Earlier years exhibit very long lags between sampling and public release - consistent with limited sequencing capacity and the absence of consolidated deposition platforms - whereas delays have shortened markedly in recent years. The fitted trend indicates a decrease of 0.782 years per calendar year (slope = −0.782 y/y), with a strong linear relationship (R² = 0.721). (D) Binned delay in recent periods. In the past five years, the mean delay from sampling to GenBank release has improved to 2.18 years. Despite this improvement, only ∼100 LASV genomes were released over the same interval, a modest number compared with the often-cited estimate of ∼300,000 human LF cases per year.

Genomic surveillance provides critical insights into LASV genetic diversity, transmission dynamics, and the geographic distribution of different LASV lineages. It is important to consider the current genomic surveillance pattern because it directly impacts our ability to understand and respond effectively to outbreaks. We downloaded all LASV sequences from NCBI Virus on 30 September 2025 and upon basic cleaning yielded 2,333 sequences. It is also important to note that LASV segments are typically submitted as separate sequences. Nigeria shows the longest sampling range, beginning in 1969 – the year of the first confirmed LF case (Figure 4A). Nigeria also exhibits a large sampling spike around 2017–2018, coinciding with a major epidemic [35]. Sampling across all countries declines markedly after 2020, likely due to diversion of surveillance resources during the COVID-19 pandemic.

Figure 4 shows the current sampling by country, based on our dataset: Nigeria 1,354 (58.0%); Guinea 458 (19.6%); Sierra Leone 305 (13.1%); Liberia 75 (3.2%); Côte d’Ivoire 39 (1.7%); Benin 31 (1.3%); Mali 25 (1.1%); Togo 6 (0.3%); Germany 2 (0.1%); Ghana 2 (0.1%); Unknown 36 (1.5%) – for a total of 2,333 sequences (Figure 4B). By host (Figure 4B), *Homo sapiens* 1,375 (58.9%) and *Mastomys natalensis* 785 (33.6%) dominate, followed by *Rodentia* (unspecified) 57 (2.4%), *Mastomys erythroleucus* 44 (1.9%), *Mastomys* (unspecified) 39 (1.7%), *Mus baoulei* 8 (0.3%), and Unknown 7 (0.3%). Additional hosts include *Hylomyscus pamfi* (6; 0.3%), *Lophuromys sikapusi* (4; 0.2%), *Capra hircus* (3; 0.1%), *Rattus rattus* (2; 0.1%), *Cavia porcellus* (2; 0.1%), and *Canis lupus familiaris* (1; <0.1%). Since the discovery of LASV in surprising new hosts, rodent sampling has been reported to be on the increase in recent years [17,18]. Guinea currently contributes the most sequences from rodents – specifically *Mastomys natalensis* – and rodent sequences sampled there massively exceeds human sequences (Figure 4B). Due to differences in LASV case number reporting among these countries [52], it is unclear if the genomic sampling distribution is representative of regional LASV incidence.

The time from sampling to sequence release has declined dramatically since the discovery of LASV in 1969 (Figure 4C). This is particularly important for our new resources, which depend on GenBank data. Early lags were as long as ∼40 years, whereas the average over the last 10 years is ∼2.67 years (Figure 4D). On average, the delay between sample collection and public release has been shrinking by about 0.78 years per calendar year (∼9.4 months less delay each year). Over a decade, this corresponds to ≈ 7.8 years less delay. A simple linear trend with collection date explains ∼72% of the variability in delay times, consistent with a strong and sustained improvement over time.

### Global real-time LASV trees

**Figure 5.**
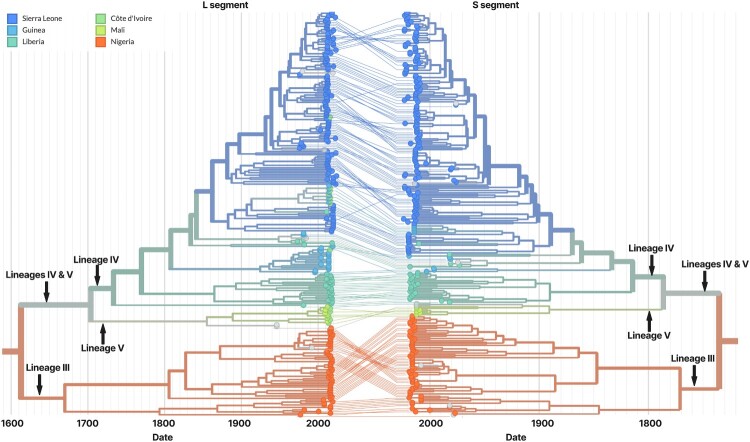
Tanglegram of LASV L- and S-segment subtrees (Lineages III and IV/V). Notes: Tips are coloured by country and branches are annotated by lineage; lines connect the same isolates across segments. Lineage V includes the Soromba strains KF478763 (L) and KF478765 (S) [27]. On the time-scaled trees, the basal split within lineage III predates the split between lineages IV and V on both segments: L: 1669-11-29 (95% CI 1665-05-04-1675-03-09); S: 1771-01-23 (95% CI 1765-02-12-1776-12-23). The IV-V split is dated L: 1703-10-31 (95% CI 1699-12-14-1709-02-05); S: 1786-05-09 (95% CI 1781-02-02-1791-08-27). These dates indicate that lineage III's major sublineages diverged earlier in time than the separation of lineages IV and V, and this ordering is consistent across both genomic segments.

To support near real-time genomic surveillance of LASV, we developed three publicly accessible, open-source phylogenetic trees within the Nextstrain platform: one each for the L segment, S segment, and the GPC gene. These trees can be viewed on either a divergence scale or an inferred time scale. Each build is continually updated with new sequence data from GenBank. All newly added sequences are automatically aligned, quality-checked, and assigned to lineage using our standardized pipeline (see Methods). These trees can be accessed at nextstrain.org/lassa/l, nextstrain.org/lassa/s, and nextstrain.org/lassa/gpc, respectively. To facilitate segmental comparison, we also implemented a tanglegram view that links individual isolates between the L and S trees (nextstrain.org/lassa/l:lassa/s). There is currently no indication of reassortment between major LASV lineages. Figure 5 shows a subtree of the tanglegram. Across both segments, the basal split within lineage III predates the separation of lineages IV and V (Figure 5). On the L segment, the most recent common ancestor (MRCA) of lineage III’s two principal sublineages is dated to 1669-11-29 (95% CI 1665-05-04–1675-03-09), whereas the IV–V MRCA is dated to 1703-10-31 (95% CI 1699-12-14–1709-02-05). On the S segment, the lineage III split is dated to 1771-01-23 (95% CI 1765-02-12–1776-12-23) versus 1786-05-09 (95% CI 1781-02-02–1791-08-27) for IV–V. The non-overlapping CIs on each segment support the ordering that lineage III’s major sublineages arose earlier.

The Nextstrain phylogenies are fully interactive and support metadata-driven views, so users can recolour the trees by sampling country, host, or assigned lineage as needed. At present, the GPC build contains more than two hundred additional sequences compared with either segment build, which gives it noticeably finer resolution for visualizing antigenic diversity and for spotting potential immune-escape trajectories. Lineage and clade labels were as described in the Methods section. In line with Whitmer et al. [28] and Daodu et al. [36], we merge IV and V because their divergence is comparable to sublineage distances within other established lineages.

As of 10 October, 2025, the builds show a consistent picture: lineage II predominates across all datasets, with IV/V forming the second largest cluster and other lineages present at much lower frequencies. In the L segment tree (n = 708), lineage II accounts for 444 genomes (62.7%), IV/V for 193 (27.3%), III for 54 (7.6%), VII for 13 (1.8%), I for 3 (0.4%), and VI for 1 (0.1%). The S segment tree (n = 784) shows a similar pattern – II at 500 (63.8%), IV/V at 197 (25.1%), III at 65 (8.3%), VII at 15 (1.9%), I at 5 (0.6%), VI at 1 (0.1%), and a single unclassified genome. The GPC tree (n = 1,142) is larger and again dominated by lineage II with 616 sequences (53.9%), followed by IV/V with 423 (37.0%), III with 70 (6.1%), VII with 22 (1.9%), I and VI with five sequences each (0.4% each), and one unclassified sequence. The unclassified sequence on the S-segment–based builds (LASV/H.sapiens-tc/NGA/2016/IRR_006), which Ehichioya et al. [25] considered novel, appeared closest to lineages I and VI; accordingly, we retained its L-segment assignment as lineage I, the better-characterized clade, pending additional data to resolve relationships among these lineages. Taken together, these totals highlight both the numerical dominance of lineage II and the advantage of the GPC for high-resolution, lineage-aware visualization.

### Live phylogeography of the LASV enabled by Nextstrain

Nextstrain enables live phylogeographic visualization of LASV, providing dynamic maps that integrate sequence data with sampling dates to trace how lineages move across space and time. In our L, S, and GPC builds, users can inspect geographic clustering of related viruses alongside the inferred direction of movement between countries, helping to identify regional transmission patterns and likely pathways of cross-border spread, including imported cases.

**Figure 6.**
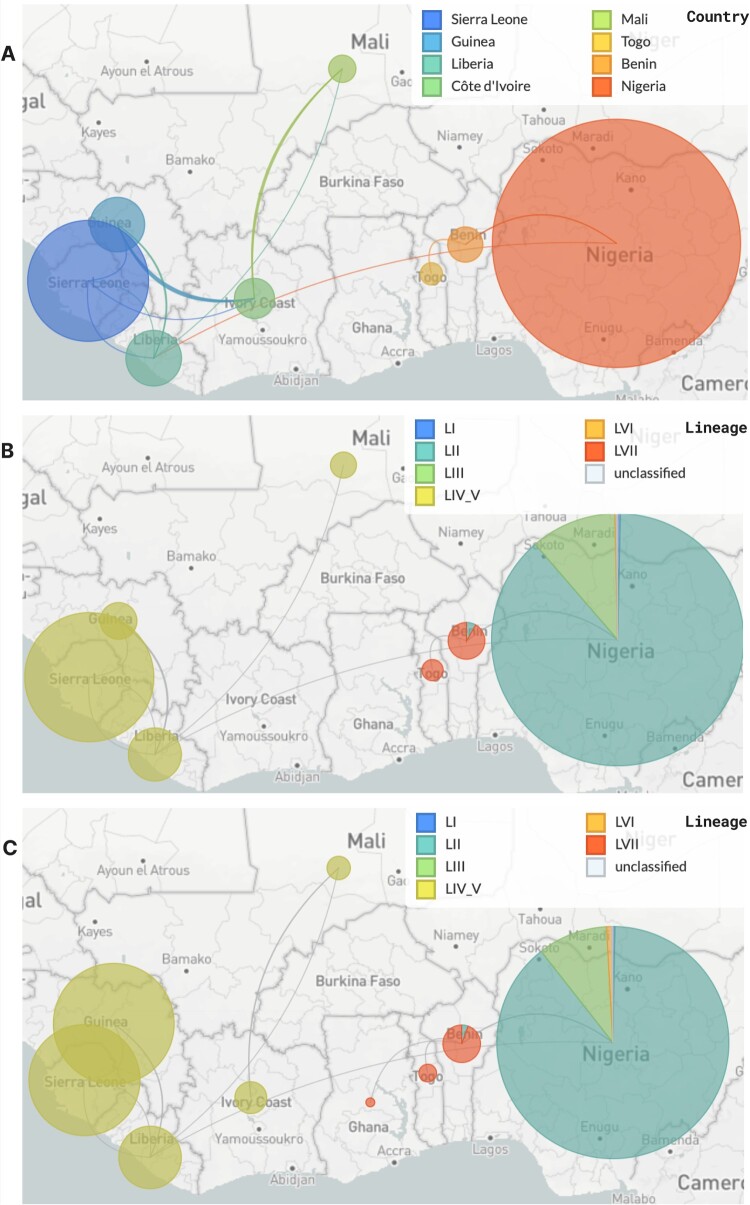
Live phylogeographic visualization of LASV from the L and S segments and the GPC gene. Note: (A) L segment (coloured by country). Inferred migration pathways highlight pronounced spatial clustering between isolates from Côte d'Ivoire and Guinea, consistent with a historical transmission link. This connection is not recovered in the S segment, likely reflecting missing Côte d'Ivoire sequences in that dataset. (B) S segment (coloured by lineage). The S map reveals a distinct connection between Mali and Liberia, indicating plausible cross-border movement within the corresponding lineage background. (C) GPC gene (coloured by lineage). The GPC map shows the greatest data density, consistent with widespread use of partial-gene sequencing targeting this immunologically relevant region. In this view, lineage VII appears to extend westward from Benin into Togo and Ghana.

Consistent with earlier work [26,53], our analyses indicate that Nigeria is the most likely ancestral state of the most recent common ancestor (MRCA). In Figures 6A–C, we inferred movement of viral lineages from Nigeria to Benin and from Nigeria to Liberia, patterns compatible with the view that viruses assigned to lineage VII (Benin) and lineage IV (Liberia and Sierra Leone) arose independently from ancestral Nigerian strains.

The GPC build further suggests a tentative westward trajectory of lineage VII from Benin to Togo and then into Ghana; this finding is uncertain because the Ghana sequence (JX845171) is short (∼949 nt of the ∼1,473 nt GPC), which reduces temporal and phylogeographic resolution. Across the segment-based maps, lineage II – typically associated with southern Nigeria – also appears in Benin, consistent with a documented cross-border importation [29]. The most likely ancestral state of the MRCA of lineages IV and V is inferred to be Liberia, consistent with Wang et al. [53].

As additional genomes are uploaded from diverse locations, these continuously updated resources will allow researchers and public-health officials to monitor transmission trends in near real time, refine hypotheses about lineage origins and routes of spread, and prioritize targeted surveillance where introductions or onward transmission are most likely.

### Tracking of important GPC mutations enabled by Nextstrain

Because it mediates entry and is the only surface-exposed viral protein, the LASV GPC is the principal target of host immunity and therapeutic monoclonal antibodies [24,54]. To enable focused surveillance, we built a GPC-specific Nextstrain phylogeny constructed independently of the remainder of the S segment, thereby avoiding signal from the nucleoprotein gene. The tree is fully interactive and can be coloured by country, lineage, or user-selected amino-acid changes.

**Figure 7.**
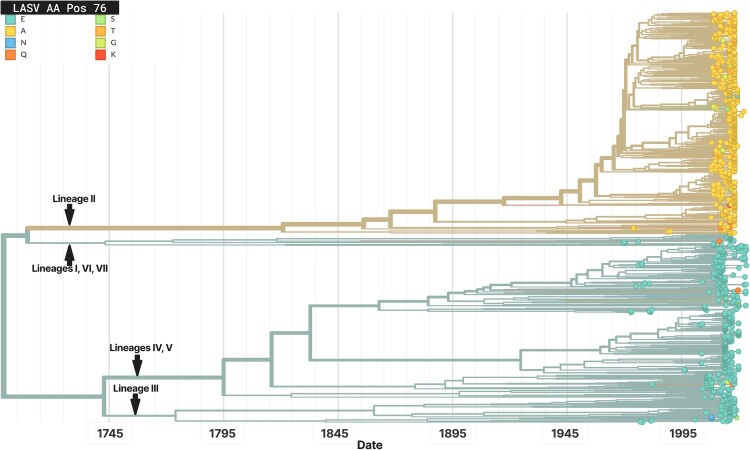
Variation at position 76 in the LASV GPC. Note: GPC-specific phylogenetic tree with branches coloured by the amino acid present at GPC position 76. Multiple residues are observed at this site; alanine (A76) - enriched in lineage II and within the mapped 25.10C epitope - is highlighted as a polymorphism of interest (structural data suggest contact at this site, while DMS shows limited escape for A76). Visualizing amino-acid variation at any GPC position enables researchers, clinicians, and public-health officials to monitor putative escape-associated changes and to adapt therapeutic or containment strategies accordingly.

As an illustration, we visualized variation at GPC position 76, a residue within the mapped epitope of the broadly neutralizing antibody 25.10C. Structural analyses indicate that 25.10C contacts E76 [55], and deep-mutational scanning (DMS) places site 76 within the 25.10C footprint [1]. In our GPC tree (Figure 7), A76 is massively prevalent in lineage II, which circulates predominantly in southern Nigeria, allowing site-specific diversity to be explored across countries and lineages. Notably, DMS data indicate that position 228 is a stronger determinant of 25.10C escape, with many substitutions reducing neutralization, whereas A76 itself does not show a strong escape signal in that assay ([1]; interactive map: https://dms-vep.org/LASV_Josiah_GP_DMS/antibody_escape.html#antibody-25-10c). Position 76 therefore remains of immunological interest from a structural perspective, but its clinical impact on 25.10C susceptibility should be interpreted cautiously and confirmed experimentally. In settings where lineage II predominates (e.g. southern Nigeria), local GPC variation – including at positions 76 and 228 – should be considered when choosing potential antibodies.

Beyond that, the platform lets users query the distribution and frequency of any amino-acid change across the phylogeny in near real time. This capability helps clinicians, researchers, and public-health officials track the emergence and spread of mutations with potential immunologic or therapeutic relevance, providing actionable context for treatment planning and outbreak preparedness.

### Rapid lassa virus lineage assignment with Nextclade

Given the ongoing seasonal epidemics of LF in West Africa and the increasing number of exported cases to non-endemic regions [20–22], there is a critical need for rapid lineage assignment and phylogenetic placement using viral genome sequences. Timely and accurate identification of circulating viral lineages is essential for guiding clinical decisions, informing public health interventions, and supporting outbreak containment efforts.

To address this, we developed three dedicated datasets for use with the Nextclade platform, tailored to the segmented nature of the LASV genome. These resources accommodate the variability in sequencing approaches, which may target different regions of the genome depending on the methodology used. The first dataset is optimized for the GPC gene, which is frequently sequenced either partially or in full due to its high diagnostic and immunological relevance. This build allows for rapid analysis and lineage assignment of GPC-derived sequences and is accessible via the following URL: https://clades.nextstrain.org/?dataset-url=https://github.com/nextstrain/lassa/tree/HEAD/nextclade_data/gpc/.

The second dataset is designed for sequences derived from the L segment, which encodes the RNA-dependent RNA polymerase. This region forms the basis of the L segment-specific build, available at: https://clades.nextstrain.org/?dataset-url=https://github.com/nextstrain/lassa/tree/HEAD/nextclade_data/l/.

The last dataset is designed for sequences derived from the S segment, which encodes the both the NP and the GPC. Used alongside the L segment Build, this would allow for interlineage reassortment analysis, available at: https://clades.nextstrain.org/?dataset-url=https://github.com/nextstrain/lassa/tree/HEAD/nextclade_data/s/.

Nextclade returns per-sequence mutation calls, lineage assignments, and the closest reference sequences to aid inference of plausible origins. The L and S Nextclade builds mirror their corresponding Nextstrain phylogenies as of 10 October 2025 – that is- the data content is same as earlier discussed. Because the Nextstrain GPC tree is substantially larger, the GPC Nextclade dataset is a curated subset (extending the panel used in Daodu et al. [36]) with additional coverage for lineages I and VI. Its current composition is: LII 480; LIV/V 195; LIII 59; LVII 16; LI 1; LVI 1; unclassified 1 (total n = 753).

Lineage assignments follow established systems used in prior LASV studies and in the LASV Nextstrain builds [17,25–29]. Consistent with prior practice [27,36], IV and V are amalgamated (IV/V) due to low inter-lineage divergence. Datasets therefore classify sequences into I, II, III, IV/V, VI, and VII.

### LASV nextclade builds and CLASV can distinguish LASV from other mammarenaviruses

Even with PCR-based detection, species identity for arenaviruses is often confirmed only after assembly and analysis [18,56,57]. Moreover, not all mammarenaviruses are pathogenic – or of comparable clinical importance – to LASV [58], creating a real clinical risk if misclassified: a false positive (calling a non-LASV mammarenavirus as LASV) could prompt unnecessary ribavirin and isolation (and false public health emergency in imported cases), whereas a false negative could delay care and enable transmission. To minimize these errors, we optimized the similarity screen in our LASV Nextclade builds by tuning a single parameter – the minimum seed-cover – so that clearly non-LASV sequences are rejected outright and bona fide LASV sequences proceed to lineage assignment. Empirically (see Methods), the optimal thresholds were 0.120 for the L segment and 0.220 for the S segment; we also used 0.22 for the GPC build. We then benchmarked performance on comprehensive datasets comprising, for the S segment, 853 LASV sequences plus representatives of 55 other mammarenaviruses leading to a total of 1096 (Figure 3; accompanying GitHub data), and for the L segment, 690 LASV sequences plus representatives of 55 other mammarenaviruses leading to a total of 916 (Figure 3; accompanying GitHub data). We evaluated three LASV Nextclade builds (L, S, and GPC) alongside our previously published LASV GPC amino-acid classifier, CLASV [36], in both its general and out-of-distribution (OOD) modes.

**Figure 8.**
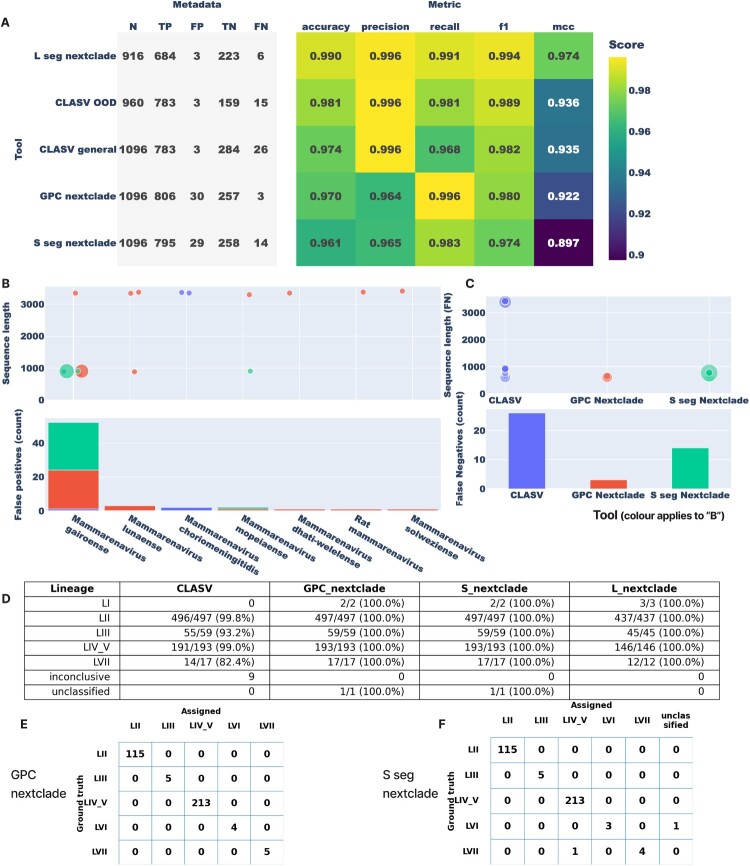
Performance of Nextclade tools and CLASV in distinguishing LASV and assigning lineages. Note: (A) Comparison of lineage-assignment performance among the Nextclade L, S, and GPC builds and CLASV, ranked by the Matthews correlation coefficient (MCC). The L-segment Nextclade build performed best (MCC = 0.974), followed by CLASV in out-of-distribution (0.936) and general (0.935) modes, the GPC build (0.922), and the S-segment build (0.897). (B) Distribution of sequence lengths among false positive records. The GPC build returned several long, variable sequences, whereas the S-segment and CLASV builds primarily flagged shorter, more uniform ones. Most false positives originated from *Mammarenavirus gairoense* (Gairo virus), with a few from *M. lunaense*, *M. choriomeningitidis*, and *M.mopeiaense*; CLASV produced the fewest false positives overall. These patterns suggest that false-positive assignments are largely restricted to Gairo virus and highlight the value of phylogenetic validation for divergent mammarenaviruses. (C) False negatives involved mostly short or incomplete sequences. In CLASV, some apparently complete S-segment (∼3 kb) sequences likely represent LASV lineages that were not included in model training. (D) Lineage-assignment accuracy on the curated mammarenavirus test set. Lineage VI was absent from the test set. All represented lineages were correctly assigned by Nextclade (100%). For CLASV, lineage I accuracy was zero (lineage I was not included in training), and nine sequences were reported as inconclusive. (E) Lineage-assignment accuracy of the GPC Nextclade build on the scaffold-excluded LASV validation set. All evaluated lineages (II, III, IV/V, VI, VII) were assigned correctly. (F) Lineage-assignment accuracy of the S-segment Nextclade build on the same scaffold-excluded validation set. Lineages II, III, and IV/V were all assigned correctly. For lineage VI, three of four sequences were assigned to lineage VI and one was returned as unclassified; for lineage VII, four of five sequences were correctly assigned and one was misclassified as lineage IV/V. These results further show that the S and GPC Nextclade builds assign LASV lineages accurately, even for sequences not used to construct the scaffold trees.

Across these experiments, all tools cleanly distinguished LASV from other mammarenaviruses. Figure 8 shows the performance of the Nextclade tools and CLASV in distinguishing LASV and assigning lineages. Using Matthews correlation coefficient (MCC) as the summary metric (Figure 8A), the L-segment Nextclade build performed best (MCC = 0.974), followed by CLASV in OOD mode (0.936) and general mode (0.935), the GPC Nextclade build (0.922), and the S-segment Nextclade build (0.897). These results indicate that the seed-cover gate effectively prevents non-LASV sequences from being misrouted into LASV lineage calls, while allowing true LASV to receive accurate lineage labels. Practically, the tuned Nextclade builds, together with CLASV, provide a conservative, clinically appropriate approach: they reduce false positives by rejecting dissimilar mammarenaviruses at the screening step and reduce false negatives by preserving high sensitivity among genuine LASV sequences.

Error analyses are shown in Figure 8B–C. In total, false positives (non-LASV misclassified as LASV) were three for the L-segment Nextclade, three for CLASV, 30 for the GPC Nextclade, and 29 for the S-segment Nextclade. In the S-segment evaluations (both S and GPC builds), most (28 for S seg Nextclade, 23 for GPC Nextclade) false positives were Gairo virus, a recently described mammarenavirus [56]. For CLASV, two of the three false positives were LCMV and one was Gairo virus. Many of these miscalled entries were short (∼1,000 nt), far below the expected length of >3,000 nt for the S segment (and well below GPC lengths of >1,200 nt), and false negatives in the S-segment tests showed the same pattern, predominantly caused by short input sequences (Figure 8C). For the L segment, all LASV false negatives (accessions GU573541–GU573546) were ∼754 nt, well below the expected ∼7 kb. By contrast, most L-segment false positives were long (7 kb in length), apparently high-quality non-LASV genomes; notably, two of the three were Luna virus, reported to be phylogenetically close to Lassa virus [56]. Taken together, these observations indicate that sequence incompleteness is the principal driver of error, whereas misclassification due to phylogenetic proximity appears limited.

This dataset therefore enabled robust testing of both CLASV and the Nextclade-based lineage-assignment builds, although several caveats must be considered. Because CLASV was trained on publicly available LASV sequences, there is a possibility that some sequences in our evaluation set overlapped with those used to train the released Random Forest (RF) model. As a consequence, partial overlap between training and evaluation data could lead to modestly inflated performance estimates. Nonetheless, the test set was sufficiently diverse to probe CLASV’s propensity towards false-positive LASV identifications and lineage calls; in this context, performance metrics that penalize false positives (precision) are particularly informative (Figure 8). CLASV’s lineage-assignment performance on unseen data has already been characterized in detail by Daodu et al. [36].

For the Nextclade-based tools, the dataset was also appropriate. Each Nextclade dataset used a single LASV reference sequence as the alignment anchor, to which all query sequences were aligned before mutation calling (see Methods). Query sequences were then placed onto a fixed, pre-computed phylogenetic reference tree annotated with clade/lineage definitions, and lineage assignment was derived from this placement; Nextclade does not fit or update any model parameters based on the input data. Consequently, the fact that some of our evaluation sequences may already be present as tips in the underlying reference tree does not introduce the same kind of train–test leakage encountered in machine-learning models.

To further demonstrate that our S-segment-based Nextclade builds generalized beyond the sequences used to construct the reference tree, we additionally evaluated them on S-segment sequences that were explicitly excluded from the tree-building and dataset-configuration process.

### LASV nextclade builds can accurately assign lineages

First, we assessed the lineage-assignment performance of the LASV Nextclade L-, S-, and GPC-based builds alongside CLASV using outputs from the curated mammarenavirus datasets described above. Ground-truth labels were taken from the corresponding Nextstrain phylogenies: the GPC Nextstrain tree provided lineage calls for evaluating the GPC and S builds and CLASV, while the L Nextstrain tree provided labels for evaluating the L build (see Methods). Records lacking a definitive lineage on these reference trees were excluded, yielding 769 sequences for the S/GPC/CLASV analysis and 643 sequences for the L analysis. The S set comprised LI (n = 2), LII (n = 497), LIII (n = 59), LIV/V (n = 193), LVII (n = 17), and one unclassified sequence. The L set comprised LI (n = 3), LII (n = 437), LIII (n = 45), LIV/V (n = 146), and LVII (n = 12).

Across these evaluations, all three Nextclade builds correctly assigned lineages for every sequence (Figure 8D). CLASV also performed strongly, with lineage-specific variation: 99.8% accuracy for lineage II, 93.2% for lineage III, 99.0% for lineages IV/V, and 82.4% for lineage VII; nine of 769 sequences were returned as inconclusive. Overall, these results indicate that the lineage-assignment capabilities of the Nextclade builds are functioning as expected and provide reliable lineage assignment across the major LASV clades, while CLASV offers high accuracy with some heterogeneity by lineage, consistent with our earlier observations and with reports that CLASV is not optimal for lineage VII classification [36]. The high performance of both tools, and the superior performance of Nextclade relative to CLASV in this setting, are expected given that Nextclade uses explicit phylogenetic placement on curated reference trees and LASV lineages are clearly phylogenetically separated. In this context, many sequences can be placed by matching to closely related sequences already represented on the tree. Lineage VI was not evaluated here because available records in NCBI Virus lacked consistent segment annotation and the lineage remains sparsely represented (for example, KT992435.1 for L and KT992425.1 for S).

Second, to obtain a more stringent assessment of lineage-assignment performance and to test whether our S-segment–based Nextclade builds generalized beyond the sequences used to construct the reference tree, we evaluated lineage calls on a GPC-based test set constructed to exclude any sequences used in the scaffold trees for the S and GPC Nextclade builds. Because the S segment encodes the GPC, the sole surface protein and a major target of neutralizing antibodies, with additional antibody responses sometimes directed against the nucleoprotein, it is the most clinically relevant segment for immunological applications. We therefore focused this scaffold-excluded validation on S and GPC. We extracted 342 GPC sequence IDs from the live GPC Nextstrain tree (the largest available LASV GPC phylogeny, of which only a subset was used earlier to construct the GPC Nextclade scaffold) that were absent from both the S- and GPC-based Nextclade datasets, downloaded the corresponding sequences from GenBank, and ran them through the GPC and S Nextclade builds. Lineage assignments were then compared against the GPC Nextstrain lineages as ground truth (Figures 8E and F).

For the GPC build, Nextclade achieved 100% concordance with the ground truth for all lineages represented in this validation set: 115 sequences in lineage II, 5 in lineage III, 213 in lineages IV/V, 4 in lineage VI, and 5 in lineage VII. For the S build, lineages II, III, and IV/V were all correctly assigned (100% concordance). For lineage VI, three of four sequences were assigned to lineage VI and one was returned as unclassified. For lineage VII, four of five sequences were correctly assigned and one was misclassified as lineage IV/V. Taken together, these results show that the LASV Nextclade builds not only perform perfectly on the curated mammarenavirus test set but also generalize with very high accuracy to an independent set of LASV sequences that were explicitly excluded from the scaffold trees, across all currently sampled LASV lineages.

### Mitigating false results by phylogenetic tree visualization

**Figure 9.**
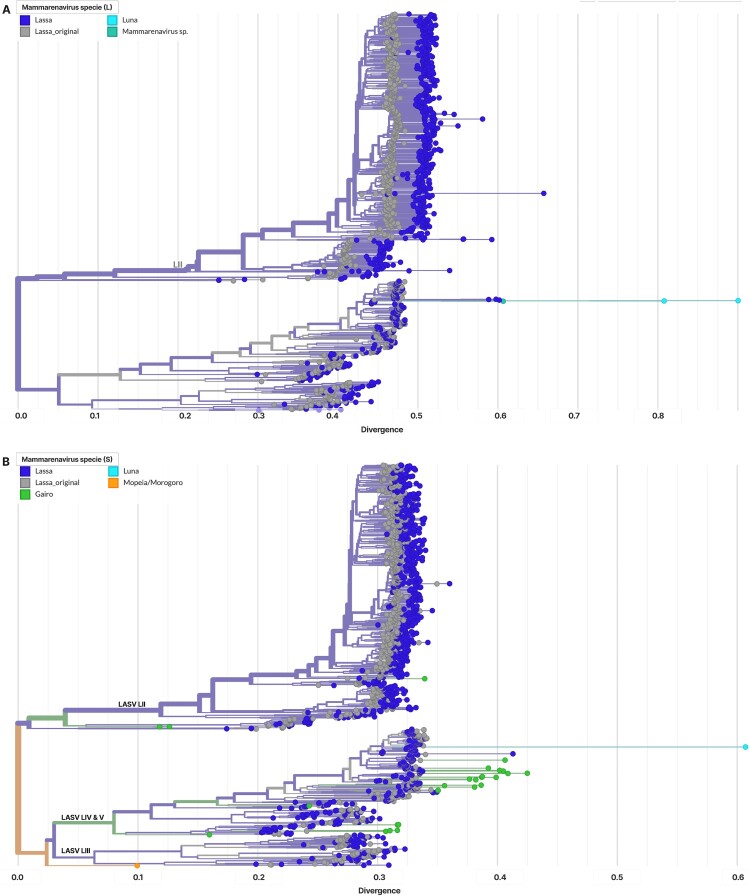
Avoiding false results through tree-structure inspection. Note: (A) Phylogenetic placement of the three false-positive sequences using the L-specific Nextclade build. LASV sequences cluster closely according to their known geographic distribution, whereas the non-LASV sequences are incorrectly classified within LASV lineage IV. Their exceptionally long branch lengths, however, clearly distinguish them from true LASV sequences, indicating a distant evolutionary origin. (B) Phylogenetic tree output from the S-segment Nextclade build. The *Mammarenavirus gaireonse* (Gairo virus) did not form a monophyletic clade but instead appeared as scattered clusters across the tree. This pattern raises important questions regarding the evolutionary relationship between Gairo virus and LASV.

Interestingly, Nextclade performs rapid phylogenetic placement of new sequences onto a reference tree, allowing users to visualize the clustering of their data in real time. To illustrate this feature, Figure 9 shows the placement of false-positive sequences from the mammarenavirus test on the L-segment (Figure 9A) and S-segment (Figure 9B) trees. False positives are readily identifiable by their exceptionally long branch lengths, which clearly distinguish them from true LASV sequences and indicate a distant evolutionary origin. In contrast, LASV sequences from the test clustered closely with their expected geographic distribution and typical evolutionary distances. This demonstrates that users can detect potential outliers or misclassifications by inspecting tree topology and branch length patterns. Interestingly, *Mammarenavirus gaireonse* (Gairo virus) [56] did not form a monophyletic clade but instead appeared as scattered clusters across the tree (Figure 9B). Although almost all available Gairo virus sequences are incomplete, this observation raises important questions regarding its evolutionary relationship with LASV. However, this relationship should be interpreted cautiously until more complete sequence data allow further investigation.

## Discussion

In this study, we present a suite of open-access resources for rapid and interactive genomic surveillance and lineage assignment of LASV, leveraging the Nextstrain and Nextclade platforms. Given the seasonal outbreaks [20], increasing frequency of exported cases [21,22] and the virus’s geographic and clinical diversity [25,26,31–33], these tools offer timely, scalable solutions for outbreak monitoring, molecular epidemiology, and public health decision-making. They also hold potential for clinical decision-making, particularly in endemic and resource-limited settings.

Our phylogenetic and phylogeographic analyses reaffirm previous findings that Nigeria is the most likely ancestral state of the most recent common ancestor (MRCA) [25,26]. Through live visualizations, we observed plausible historical transmission pathways from Nigeria to Benin and Liberia, and a potential westward spread into Ghana via Togo (Figure 6). These patterns support the hypothesis that lineages IV and VII emerged independently from ancestral Nigerian strains [53]. The detection of lineage II in the Republic of Benin suggests cross-border transmission from southern Nigeria [29] – a plausible scenario given the geographic proximity and relatively unrestricted human movement between the two countries. These phylogeographic insights provide both historical context and concurrent relevance for understanding LASV transmission dynamics in West Africa and for tracking exported cases globally.

The implementation of segment-specific phylogenetic trees – including separate builds for the L segment, S segment, and GPC gene – addresses major challenges in arenavirus genomics, such as reassortment and recombination. The tanglegram view developed for cross-segment comparison revealed that sublineages within lineage III diverged earlier than those in lineages IV and V (Figure 5), raising questions about the current nomenclature of LASV lineages. Whitmer et al. [28] previously grouped lineages IV and V together based on genetic distances comparable to those observed among recognized sublineages, a convention also followed in Daodu et al. [36] and in this study. Accordingly, we use this grouping to maintain consistency with prior literature rather than to propose a revised lineage framework, unless and until formal reclassification is undertaken. Nevertheless, the early divergence observed within lineage III broadly supports revisiting the current nomenclature. The independently constructed GPC tree offers higher-resolution tracking of genetic diversity within this immunologically and therapeutically significant region.

We also demonstrate the potential clinical and epidemiological utility of mutation tracking through live visualization of key GPC positions. For example, the A76 polymorphism – implicated in reduced binding of monoclonal antibody 25.10C [55] and discussed in DMS analyses [1] – is readily traceable on the tree and is highly prevalent in lineage II (Figure 7). Its frequency in southern Nigeria suggests potential regional constraints on some antibody therapies and emphasizes the value of geographically tailored strategies. The same framework extends to other clinically relevant mutations, including putative immune-escape or antiviral-resistance substitutions. Furthermore, because antibody-based therapies often perform best when derived from individuals infected with closely related LASV lineages [31,32], live lineage identification (Figure 8) – including at point of care (Figure 1) – can inform treatment selection. In such scenarios, convalescent plasma or monoclonal antibodies sourced from donors infected with similar or matching lineages may offer improved neutralization and therapeutic benefit. Current limitations to clinical implementation, especially in endemic regions, may include challenges related to sequence generation, such as sequencing turnaround time and infrastructure constraints. However, these barriers are expected to ease as technologies and resources continue to advance.

To democratize access to LASV lineage information for public-health use, we developed three dedicated Nextclade builds – GPC, S, and L – that accept both complete and partial sequences. We evaluated their ability to distinguish LASV from other mammarenaviruses, thereby minimizing false positives and false negatives (Figure 8). CLASV [36] also performed well at selectively identifying LASV sequences; in particular, its out-of-distribution (OOD) mode, implemented with a random-forest classifier, was effective at flagging non-LASV inputs, a capability that could be useful in future genomics workflows. For routine lineage assignment of new genomes, we recommend using the S and GPC Nextclade builds where possible, as these were most extensively validated, and using the L Nextclade build when only L-segment data are available or as an additional cross-check. Applying multiple segment-specific builds to the corresponding segments of a full LASV genome increases confidence and can help detect inter-lineage reassortment when segment-level calls disagree. To further reduce the risk of misclassifying other mammarenaviruses as LASV, CLASV can be run alongside as an orthogonal check. For immunotherapy-related questions, prioritizing the GPC Nextclade build and CLASV is appropriate, given the immunological centrality of GPC [24,54]. In practice, our tools assign lineages accurately for lineages II, III, IV/V, and VII. Lineages VI and I are not currently recommended for routine interpretation, because these lineages remain sparsely sampled and were not robustly evaluated in our analyses.

We tested the platform's accuracy (Figure 8) and found that the few false results are mostly driven by incomplete sequences. It is therefore important to use high-quality, adequately long genome sequences. Meanwhile, false results can be further mitigated by visualizing the tree provided by the Nextclade builds (Figure 9). True LASV sequences typically fall within the established genetic distances on the tree, whereas non-LASV or highly divergent inputs tend to appear on unusually long branches or outside expected clades. Interestingly, Gairo virus – a relatively new mammarenavirus that is also carried by *Mastomys natalensis* [56] – does not form a monophyletic clade on the S tree. This raises the hypothesis that Gairo virus may represent diverged LASV lineages. To evaluate this hypothesis, expanded, high-quality sequencing of Gairo virus (and related hosts) is needed to clarify its phylogenetic placement and relationship to LASV. In line with Whitmer et al. [28], we also advocate revisiting LASV lineage definitions. Overall, this work emphasizes the value of publicly available, reproducible genomic-surveillance platforms for managing endemic and emerging pathogens. The LASV-specific Nextstrain and Nextclade resources described here facilitate rapid and accurate genomic interpretation, with direct implications for clinical care, outbreak investigation, and therapeutic development. As LASV sequencing expands – both within endemic regions and via exported-case reports – these tools, which we intend to maintain and update, are well positioned to support live tracking, inform risk assessments, and strengthen global outbreak preparedness.

A pressing limitation of this study – and of our tools – is the current sampling pattern for LASV. Genomic inferences are only as good as the background data. LASV sampling appears seriously uneven across locations and hosts and is generally under- sampled (Figure 4A-B). Also, the sampling-to-sub-mission lag remains excessive (Figure 4C-D), averaging >2 years in the past five years. Given the severity of LASV, this is a danger to global public health. We urgently call on public-health authorities to address data-sharing barriers and accelerate timely deposition of high-quality sequences to GenBank and other public repositories.

## Clipart

Cliparts from open sources: https://freesvg.org/oak-tree-vector-image, https://freesvg.org/sars-cov2-virus, https://www.clipsafari.com/clips/o274545-dna-circle, https://www.clipsafari.com/clips/o241121-computer-icon, https://openclipart.org/detail/344106/social-distancing-2m-gap, https://openclipart.org/detail/308893/intensive-care, https://freesvg.org/hospital-icon, https://openclipart.org/detail/126391/docteur-doctor, https://freesvg.org/traditional-light-bulb-icon-vector-clip-art, https://openclipart.org/detail/214209/tube-sample, https://openclipart.org/detail/307233/symbolic-dna-sequencing#google_vignette, https://freesvg.org/curved-arrow.

## Code availability

The main, regularly updated repository is available at: https://github.com/nextstrain/lassa. A frozen snapshot containing the scripts, configuration files, and data underlying this manuscript is available at: https://github.com/JoiRichi/lasv_nextstrain_nextclade_extra_data.
